# Real-World Treatment Patterns and Outcomes of Intraluminal Ablative Therapies in Noninvasive Urethral Carcinoma: A National Cancer Database Analysis

**DOI:** 10.3390/curroncol33010045

**Published:** 2026-01-14

**Authors:** Eusebio Luna Velasquez, Vatsala Mundra, Renil S. Titus, Jiaqiong Xu, Carlos Riveros, Dharam Kaushik, Amar Singh, Suran Somawardana, Christopher J. D. Wallis, Raj Satkunasivam

**Affiliations:** 1 Department of Urology, Houston Methodist Hospital, Houston, TX 77030, USA; eusebioluna54@gmail.com (E.L.V.); rsatkunasivam@houstonmethodist.org (R.S.); 2 Center for Health Data Science and Analytics, Houston Methodist Hospital, Houston, TX 77030, USA; 3 Department of Bioengineering, Rice University, Houston, TX 77030, USA; 4 Division of Urology and Surgical Oncology, Department of Surgery, Princess Margaret Cancer Centre, University Health Network, University of Toronto, Toronto, ON M5S 1A1, Canada; 5 Division of Urology, University of Toronto, Toronto, ON M5S 1A1, Canada; 6 Division of Urology, Mount Sinai Hospital, Toronto, ON M5T 3L9, Canada

**Keywords:** non-invasive urethral carcinoma, urothelial cancer, urethrectomy, treatment patterns

## Abstract

**Objective**: To evaluate treatment patterns, predictors of treatment selection, and overall survival (OS) in patients with noninvasive (Ta–Tis) urothelial carcinoma of the urethra (UUC). **Patients and Methods:** We conducted a retrospective cohort study of adults diagnosed with noninvasive UUC (stage Ta or Tis, N0, M0) between 2018 and 2021 using the National Cancer Database. Patients were categorized into prostatic and non-prostatic urethral cohorts. Treatment groups included endoluminal ablation alone, ablation combined with topical intraluminal therapy, urethrectomy, and no subsequent treatment. Multinomial logistic regression was used to identify predictors of treatment selection. The OS was assessed using Kaplan–Meier and multivariable Cox regression, with separate models for each anatomical cohort. **Results:** A total of 436 patients were included (185 non-prostatic, 251 prostatic); 91.9% (*n* = 401) were male. Ablation alone was the most common treatment in both cohorts (non-prostatic: 57.3%; prostatic: 62.6%), followed by urethrectomy (non-prostatic: 21.1%) and ablation plus topical therapy (prostatic: 20.3%). In the non-prostatic cohort, high-grade histology (OR 15.15; 95% CI, 3.82–60.04) and Tis stage (OR 3.27; 95% CI, 1.10–9.69) were associated with increased odds of urethrectomy. In the prostatic cohort, high-grade histology was associated with increased use of urethrectomy (OR 59.29; 95% CI, 4.61–763.17) and ablation plus topical therapy (OR 3.09; 95% CI, 1.21–7.90). Tis stage was also associated with ablation plus topical therapy (OR 2.53; 95% CI, 1.14–5.62). This treatment approach was associated with improved OS compared with ablation alone (HR 0.18; 95% CI, 0.05–0.60; *p* = 0.005). **Conclusions:** Treatment selection differed substantially by tumor location, stage, and grade, reflecting both treatment selection in noninvasive UUC varied by tumor location, grade, and stage. In prostatic tumors, ablation plus topical therapy was associated with improved survival, supporting its role as a risk-adapted, organ-sparing strategy in selected patients.

## Simple Summary

In this study of patients with early-stage urethral cancer, we found that treatment choices were influenced by tumor location and how aggressive it appeared under the microscope. Patients treated at academic hospitals were more likely to undergo surgery. In men with cancer in the part of the urethra near the prostate, combining ablation with topical treatments was linked to better survival compared to ablation alone. These results suggest that some patients with early-stage urethral cancer may benefit from less invasive, organ-sparing approaches when chosen carefully based on their individual risk.

## 1. Introduction

Primary urethral cancer is a rare malignancy, accounting for less than 1% of all cancers worldwide. Urethral urothelial carcinoma (UUC) comprises less than 1% of all urothelial carcinomas [1,2]. At presentation, most patients exhibit symptoms consistent with advanced disease, and consequently, the existing literature largely focuses on the management and outcomes of invasive stages [3,4]. In contrast, data on early, noninvasive disease, specifically carcinoma in situ (Tis) and noninvasive papillary tumors (Ta), remain limited.

Management guidelines from the National Comprehensive Cancer Network (NCCN) and European Association of Urology (EAU) provide some direction for early-stage disease. The NCCN recommends transurethral resection (TUR) as the initial treatment for noninvasive urethral cancer, followed by Bacillus Calmette-Guérin (BCG) therapy in selected cases of Ta–T1 [5]. The EAU similarly recommends TUR for prostatic urethral Ta–T1 lesions but advises partial or total urethrectomy for comparable-stage lesions in the membranous urethra. For distal urethral tumors staged as Ta–T2, the EAU favors urethrectomy while also acknowledging urethra-sparing strategies [6].

Emerging retrospective evidence suggests that endoscopic approaches may have a role in the management of early-stage disease. Prior retrospective studies on non-invasive (Ta and Tis) urethral urothelial carcinoma are limited by small sample sizes, heterogeneous patient populations, and insufficient granularity regarding contemporary minimally invasive and topical therapies [7,8,9].

In this retrospective cohort study, we evaluated contemporary treatment patterns and outcomes among patients with non-invasive (Ta and Tis) urothelial carcinoma of the urethra, comparing different therapeutic approaches.

## 2. Methods

This retrospective cohort study used data from the National Cancer Database (NCDB), a nationwide oncology registry jointly maintained by the American College of Surgeons and the American Cancer Society. The NCDB captures standardized demographic, clinical, treatment, and outcome data from more than 1500 Commission on Cancer–accredited facilities across the United States, representing approximately 70% of newly diagnosed malignancies nationwide [10]. As the NCDB contains only de-identified patient records, this study was exempt from institutional review board (IRB) approval.

### 2.1. Study Population

**Figure 1:**
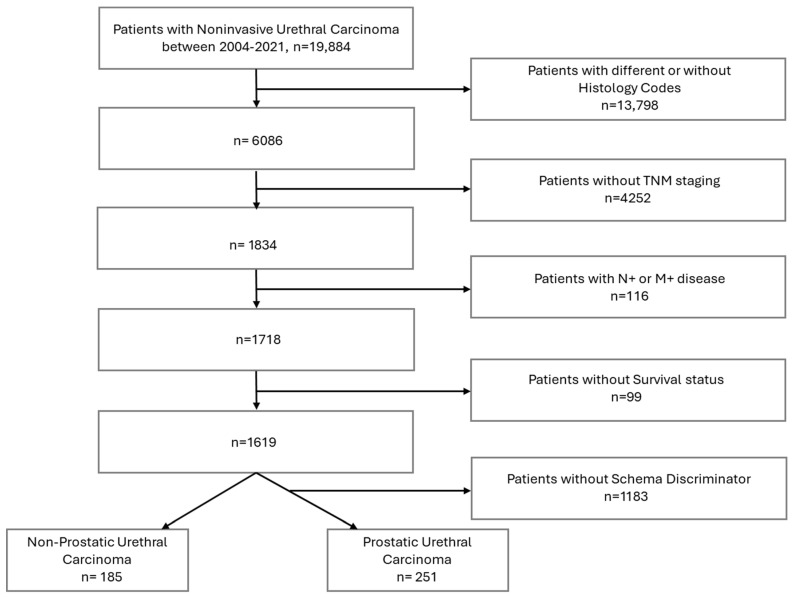
Cohort Selection for Patients with Noninvasive Urethral Carcinoma from the NCDB (2018–2021).

Adult patients (≥18 years) diagnosed between 2018 and 2021 with UUC were identified using ICD-O-3 topography code C68.0. Histology was restricted to urothelial carcinoma using ICD-O-3 histology codes 8120/2, 8120/3, 8131/3, and 8050/3. Eligible patients had clinical stage Ta or Tis disease, without nodal involvement (N0) or distant metastases (M0). Given that anatomical location has biological behavior, prognosis, and therapeutic implications, patients were stratified into prostatic and non-prostatic cohorts [5]. Prostatic urethral tumors were defined as NCDB Schema ID 00633 with Schema Discriminator 1 code 2; all others were considered non prostatic (Figure 1).

### 2.2. Exposure and Covariates

Treatment groups were defined according to surgical and therapeutic interventions recorded in the NCDB. Patients were classified as having received endoluminal ablative procedures alone (endoscopic laser or electrocautery destruction), ablation combined with topical intraluminal therapy (including bacillus Calmette–Guérin [BCG] or intraluminal chemotherapy), topical therapy alone, or urethrectomy (partial, total, or not otherwise specified). Due to the small number of patients treated with topical therapy alone, these cases were merged with the ablation plus topical group for analysis. The no subsequent treatment group comprised patients for whom no local or systemic therapy was recorded following diagnosis and who were managed with observation alone.

Covariates included age at diagnosis, sex, and race (White, Black, or other). Clinical variables comprised the Charlson–Deyo comorbidity score (CDS; 0, 1, 2, or ≥3), insurance status (private, Medicaid, Medicare, or unknown), clinical T-stage (Ta or Tis), tumor grade (low, high, or unknown), and tumor size (<2 cm or ≥2 cm). Facility-level characteristics included institution type (academic vs. non-academic) and geographic setting (urban vs. rural). Categorical variables with missing data were recoded as “unknown” to preserve analytic completeness.

### 2.3. Outcomes

The primary outcome was to identify clinical, demographic, and institutional factors associated with the selection of treatment modality. Secondary outcomes included assessment of temporal trends in treatment patterns by anatomical cohort and evaluation of overall survival (OS), defined as the time from diagnosis to death or last follow-up.

### 2.4. Statistical Analysis

Baseline characteristics were summarized according to the anatomic location of disease (prostatic vs. non-prostatic urethra). Continuous variables were reported as mean ± standard deviation (SD) or median with interquartile range (IQR), based on distribution. Categorical variables were summarized as absolute numbers and percentages. Group comparisons for categorical variables were conducted using the Chi-square test, and continuous variables were analyzed using the independent *t*-test or the Mann–Whitney U test, as appropriate. Predictors of treatment selection were evaluated using multinomial logistic regression, with ablation alone as the reference category. Separate models were constructed for the prostatic and non-prostatic cohorts, incorporating demographic, clinical, tumor-specific, and institutional covariates.

To assess temporal trends in treatment selection, a stacked histogram was generated to display yearly proportions of each treatment modality. The significance of temporal trends was evaluated using the Cochran–Armitage test.

Overall survival was evaluated using multivariable Cox proportional hazards regression, with separate models for prostatic and non-prostatic cohorts. The proportional hazards assumption was verified using Schoenfeld residuals. Kaplan–Meier curves were generated to visualize OS by treatment group within each cohort. All analyses were performed in Stata version 17 (StataCorp LLC., College Station, TX, USA), and statistical significance was defined as a two-sided *p* < 0.05 [11].

## 3. Results

A total of 436 patients with noninvasive UUC were identified between 2018 and 2021 (Figure 1, Table 1). Men comprised 91.9% of the cohort (*n* = 401). The cohort included 185 patients (42.4%) with non-prostatic tumors (female: *n* = 35 [18.9%], male: *n* = 150 [81.1%]) and 251 patients (57.6%) with prostatic tumors. The mean age was 71.7 ± 11.3 years overall, with patients in the prostatic cohort being older (73.4 ± 10.2 years) than those in the non-prostatic subgroup (69.4 ± 12.3 years). Most patients were White (91.3%, *n* = 398), followed by Black (6.7%, *n* = 29) and other races (2.1%, *n* = 9). The majority had a Charlson–Deyo score (CDS) of 0 (61.9%, *n* = 270) and were treated at non-academic centers (61.2%, *n* = 267).

Ta stage disease was more common than Tis (68.8% vs. 31.2%). Prostatic noninvasive tumors were more frequently Tis (37.1%, *n* = 93) compared with non-prostatic tumors (23.2%, *n* = 43). High-grade tumors accounted for 39.0% (*n* = 170), low-grade tumors for 37.6% (*n* = 164), and grade was unknown in 23.3% (*n* = 102). The most frequently used treatment was ablation alone (60.3%, *n* = 263), followed by ablation plus topical therapy (15.1%, *n* = 66), surgical urethrectomy (14.4%, *n* = 63), and no subsequent treatment (10.1%, *n* = 44). Ablation plus topical therapy was more common in prostatic tumors (20.3%, *n* = 51) than in non-prostatic noninvasive lesions (8.1%, *n* = 15), whereas urethrectomy was more frequent in non-prostatic tumors (21.1%, *n* = 39) compared with prostatic tumors (9.6%, *n* = 24) (Table 1).

### 3.1. Primary Outcome: Predictors of Treatment Selection

Multinomial logistic regression was performed separately for non-prostatic and prostatic noninvasive UUC cohorts, using ablation alone as the reference category (Table 2 and Table S1). In both cohorts, treatment at non-academic facilities was associated with significantly lower odds of urethrectomy (non-prostatic: OR 0.23, 95% CI 0.08–0.60, *p* = 0.003; prostatic: OR 0.21, 95% CI 0.06–0.67, *p* = 0.008). Among patients with prostatic noninvasive UUC, high-grade histology was independently associated with increased odds of both urethrectomy (OR, 59.29; 95% CI, 4.61–763.17; *p* = 0.002) and ablation plus topical intraluminal therapy (OR, 3.09; 95% CI, 1.21–7.90; *p* = 0.018). Similarly, Tis stage in this cohort was associated with higher odds of receiving ablation plus topical therapy (OR 2.53, 95% CI 1.14–5.62, *p* = 0.023). Increasing comorbidity burden, as measured by the Charlson–Deyo Score (CDS), was linked to lower likelihood of receiving combination therapy (CDS 1: OR, 0.33; 95% CI, 0.11–0.96; *p* = 0.042; CDS 3: OR, 0.22; 95% CI, 0.05–0.91; *p* = 0.036). In the non-prostatic cohort, both high-grade histology (OR, 15.15; 95% CI, 3.82–60.04; *p* < 0.001) and Tis stage (OR, 3.27; 95% CI, 1.10–9.69; *p* = 0.033) were significantly associated with increased odds of undergoing urethrectomy.

### 3.2. Secondary Outcomes

#### 3.2.1. Temporal Trends of Treatments

**Figure 2:**
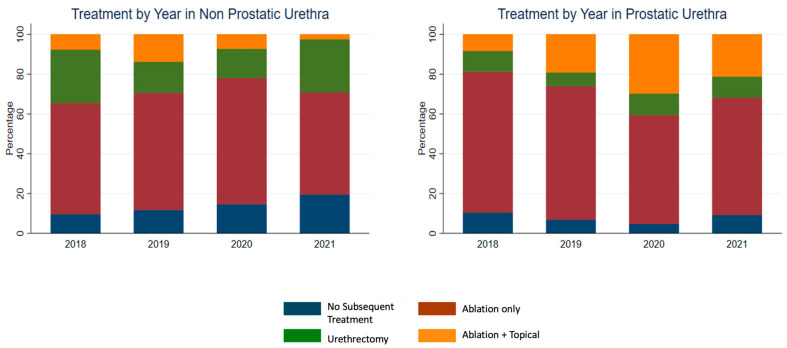
Treatment Trends by Year for Noninvasive Non-Prostatic and Prostatic Urethral Carcinoma (2018–2021). Stacked bar graphs illustrate the distribution of treatment modalities over time among patients with noninvasive non-prostatic (**left panel**) and prostatic (**right panel**) urethral carcinoma, based on National Cancer Database data from 2018 to 2021. Treatment categories include: no subsequent treatment, ablation only, urethrectomy, and ablation combined with topical intraluminal therapy.

From 2018 to 2021, ablation alone remained the most frequently used treatment in both cohorts (Figure 2). In the non-prostatic urethral carcinoma cohort, the distribution of treatment strategies demonstrated no significant temporal changes. The annual use of ablation ranged from 51.2% to 63.4%, with modest year-to-year variation (*p*  = 0.52). Rates of urethrectomy fluctuated slightly but did not follow a significant trend (*p* = 0.15), and the use of ablation combined with topical intraluminal therapy remained infrequent, with no significant increase over time (*p* = 0.27; Table 3, Figure 2). Similarly, in the prostatic cohort, overall treatment patterns remained stable during the study period. While ablation monotherapy continued to predominate, there was a non-significant increase in the use of ablation plus topical therapy, rising from 8.3% in 2018 to 21.2% in 2021 (*p* = 0.12; Table 3, Figure 2).

#### 3.2.2. Overall Survival Analysis

In the prostatic UUC cohort (Table 4 and Table S2), treatment with ablation plus topical therapy was associated with improved OS compared with ablation alone (HR 0.18, 95% CI 0.05–0.60, *p* = 0.005). Additionally, in this cohort, increasing age was independently associated with higher all-cause mortality (HR 1.07 per year, 95% CI 1.03–1.11, *p* < 0.001).

In the non-prostatic UUC cohort, tumor size ≥ 2 cm was associated with worse OS (HR 7.77, 95% CI 1.26–47.73, *p* = 0.027) (Table 4).

**Figure 3:**
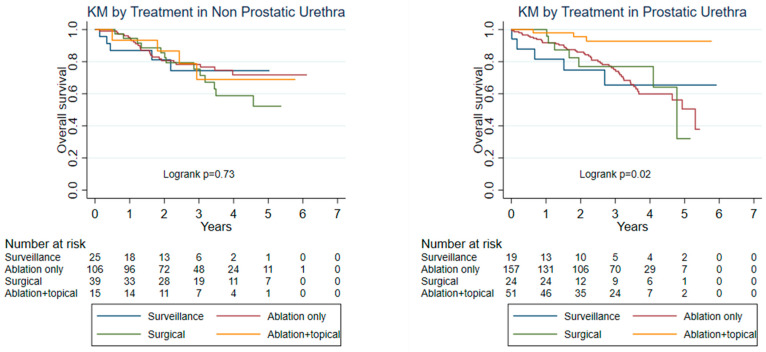
Kaplan–Meier Curves for Overall Survival by Treatment Modality in Non-Prostatic and Prostatic Urethral Carcinoma. Kaplan–Meier curves depict overall survival (OS) stratified by treatment modality in patients with non-invasive urothelial carcinoma of the non-prostatic urethra (**left**) and prostatic urethra (**right**). Treatment groups include surveillance, ablation only, surgical treatment (urethrectomy), and ablation combined with intraluminal topical therapy. Log-rank tests were used to compare survival distributions across treatment groups.

Across both cohorts, greater comorbidity burden was associated with worse OS. In the prostatic cohort, CDS 2 (HR 3.54, 95% CI 1.49–8.42, *p* = 0.004) and CDS 3 (HR 2.71, 95% CI 1.33–5.49, *p* = 0.006) were significantly associated with reduced OS compared with CDS 0. In the non-prostatic cohort, CDS 1 (HR 2.66, 95% CI 1.29–5.45, *p* = 0.008) was associated with worse OS compared with no comorbidities. Kaplan–Meier curves illustrate the survival patterns across treatment groups for both cohorts (Figure 3).

## 4. Discussion

In this multicenter, retrospective cohort study of patients with noninvasive, localized urethral urothelial carcinoma (Ta–Tis, N0, M0), treatment selection varied significantly by tumor location, grade, and stage. Management at non-academic centers was associated with lower odds of surgical intervention in both anatomical cohorts. Among patients with non-prostatic disease, high-grade histology and Tis stage were associated with increased likelihood of undergoing urethrectomy. In the prostatic cohort, high-grade histology was linked to greater use of both urethrectomy and ablation combined with topical intraluminal therapy. Tis stage in this cohort was also associated with higher odds of receiving ablation plus topical therapy, a treatment modality that was further associated with improved overall survival.

These treatment patterns likely reflect underlying anatomical factors that influence the feasibility and effectiveness of local therapies. In the non-prostatic urethra, limited surface continuity with the bladder may reduce the exposure of lesions to intravesical agents, diminishing the effectiveness of topical intraluminal therapy and leading to a greater reliance on surgical extirpation—particularly for high-grade or Tis lesions. In contrast, the prostatic urethra maintains direct continuity with the bladder, allowing more consistent and effective exposure to intravesical therapies following endoscopic ablation [12,13].

Among patients with non-prostatic tumors, urethrectomy was more frequently employed in those with high-grade or Tis lesions. The European Association of Urology (EAU) guidelines support urethrectomy for proximal non-prostatic Ta-T1 urethral tumors, although urethra-sparing approaches may be appropriate in carefully selected cases [6]. These findings are consistent with prior studies, including work by Karnes et al., who advocate for penile-preserving urethrectomy in appropriately staged tumors, and Dalbagni et al., who demonstrated that tumor location, grade, and stage are key prognostic factors that help guide surgical versus conservative management [14,15].

For prostatic urethral tumors, endoluminal ablation alone or in combination with topical therapy was the most common approach, consistent with NCCN and EAU guidelines recommending TUR followed by BCG or intraluminal chemotherapy for Ta–T1 prostatic lesions [5,6]. Prior retrospective series by Palou et al. and Gofrit et al. have demonstrated favorable oncologic outcomes with such regimens, reporting bladder preservation rates up to 70% and recurrence-free survival approaching 90% [12,16,17]. Similarly, Taylor et al. observed durable disease control in the majority of patients in their retrospective cohort study, with only 28% requiring cystectomy after initial BCG at a median follow-up of 7.5 years [18]. Collectively, these findings reinforce the role of conservative multimodal therapy in appropriately selected patients with noninvasive prostatic urethral carcinoma.

Treatment at non-academic centers was associated with decreased odds of urethrectomy across tumor subtypes. While this finding may reflect greater use of organ-preserving strategies aligned with clinical guidelines, interpretation warrants caution. Prior studies in bladder cancer have demonstrated that patients treated at community facilities are less likely to receive cancer-directed therapy for early-stage disease, raising concerns about potential undertreatment [19]. Additionally, data from the RARECAREnet database suggest that centralization of care for rare cancers enhances standardization and reduces variability, particularly in settings with limited clinical experience [20]. In contrast, the increased use of urethrectomy observed at academic institutions may reflect broader access to multidisciplinary expertise, including input from urologic oncologists and genitourinary pathologists, which may support more aggressive surgical decision-making. These findings are consistent with those of Stone et al., who reported higher rates of radical surgery in academic centers, although their analysis did not stratify by tumor stage, an important distinction addressed in the present study [21].

Moreover, while Morra et al. identified female sex as an independent predictor of bi-/trimodal therapy in patients with invasive urethral carcinoma, our study did not observe a significant association between sex and treatment modality. However, direct comparison is limited by important methodological differences. Morra et al. included only patients with invasive (≥T1) disease across heterogeneous histologic subtypes and analyzed treatment using a binary classification (single vs. bi-/trimodal therapy) [22]. In contrast, our study focused exclusively on noninvasive (Ta–Tis) urothelial carcinoma and examined treatment selection across distinct modalities. Differences in disease severity and histologic uniformity may contribute to the absence of sex-based disparities in our cohort.

Although no prior studies have formally evaluated national treatment trends in noninvasive urethral carcinoma, our findings suggest increasing adoption of guideline-concordant, risk-stratified care. We observed a modest decline in urethrectomy use in both cohorts and a corresponding increase in organ-preserving strategies, particularly ablation combined with intraluminal therapy in the prostatic cohort. These trends align with current NCCN and EAU recommendations that support conservative management for early-stage prostatic and non-prostatic urethral tumors [5,6].

Overall survival in our cohort was significantly influenced by both treatment modality and patient-level characteristics. Among patients with prostatic noninvasive UUC, the combination of endoluminal ablation and topical intraluminal therapy was associated with improved OS compared with ablation alone, suggesting a potential oncologic benefit of multimodal conservative treatment in selected patients. These findings are consistent with prior series demonstrating favorable oncologic outcomes in appropriately selected patients managed with conservative strategies. Taylor et al. reported durable long-term responses in patients with superficial prostatic urethral carcinoma treated with transurethral resection and BCG, with most avoiding progression or cystectomy [18].

Increasing age and higher Charlson–Deyo comorbidity scores emerged as independent predictors of decreased OS, underscoring the importance of patient-level factors in oncologic outcomes. These findings are consistent with a prior NCDB-based study by Sui et al., in which both advanced age and comorbidity were independently associated with reduced OS among patients with urethral carcinoma, irrespective of disease stage [23]. Similarly, Champ et al., using a SEER-based cohort, identified older age as a key predictor of poorer outcomes in patients with nonmetastatic urethral carcinoma [24].

In contrast to prior studies of invasive disease, our analysis of noninvasive urothelial UUC revealed no race- or sex-based disparities in outcomes. Wenzel et al., in retrospective analyses of patients with invasive urethral cancer (≥T1 stage), reported that Hispanic patients had a significantly higher risk of cancer-specific mortality (CSM) compared to Caucasians, and that female sex was associated with higher 5-year CSM, particularly in non-metastatic, nonurothelial disease [25,26]. The discrepancy between these findings and ours likely reflects fundamental biological and clinical differences between noninvasive and invasive disease, including stage at presentation and histologic subtype. Additionally, our use of OS rather than CSM may have attenuated sociodemographic associations, as OS is influenced by comorbidities and non-cancer causes of death, factors that may obscure cancer-specific disparities in a population with generally favorable prognosis.

This study represents one of the largest population-based analyses to date focused exclusively on noninvasive urothelial carcinoma of the urethra, offering a comprehensive evaluation of contemporary treatment patterns and associated oncologic outcomes. By restricting the cohort to urothelial histology, we reduced biological heterogeneity, thereby enhancing the validity of comparative analyses and providing clinically relevant insights for managing this rare malignancy.

Nonetheless, several limitations warrant consideration. The retrospective design of the study inherently limits causal inference and introduces both selection and coding bias. The NCDB is not a population-based registry and primarily captures data from Commission on Cancer–accredited institutions, which may differ systematically from non-academic or international settings, limiting generalizability. Moreover, the database includes only information from the index procedure and initial treatment course, without capturing local recurrence, disease progression, or development of metastases. Margin status was also inconsistently reported across treatment modalities, particularly for minimally invasive approaches, reflecting known variability in documentation and coding. In addition, several clinically relevant variables, including performance status, tumor multifocality, treatment response, and use of maintenance intravesical therapy, were unavailable. Although this study represents one of the largest reported cohorts in this disease space, the modest sample size may have limited our ability to detect more nuanced associations. Finally, the absence of disease-specific survival data restricts interpretation of cancer-related outcomes. Despite these limitations, the findings provide meaningful insights into national practice patterns and survival correlates in a rare and understudied malignancy.

## 5. Conclusions

In this large, national cohort of patients with noninvasive urothelial carcinoma of the urethra, treatment selection was significantly influenced by tumor location, stage, grade, and facility type. High-grade and Tis lesions in non-prostatic locations were more commonly managed surgically, whereas in the prostatic urethra, combination endoluminal ablation with topical intraluminal therapy was more frequently employed and associated with improved overall survival. These findings support the role of risk-adapted, organ-preserving strategies in appropriately selected patients and highlight the value of national registry data in informing treatment decisions for rare genitourinary malignancies.

## Figures and Tables

**Table 1:** Patient Demographics, Tumor Characteristics, and Treatment Patterns Among Individuals with Noninvasive Urothelial Carcinoma of the Urethra, Stratified by Anatomic Location (Non-Prostatic vs. Prostatic).

	Urethral Urothelial Carcinoma			
	Total	Non-Prostatic	Prostatic	*p*-Value
	*n* = 436	*n* = 185	*n* = 251	
*Age*	71.69 ± 11.32	69.35 ± 12.33	73.42 ± 10.19	<0.001
*Sex*				<0.001
Female	35 (8.03)	35 (18.92)	0 (0.00)	
Male	401 (91.97)	150 (81.08)	251 (100.00)	
*Race*				<0.001
White	398 (91.28)	157 (84.86)	241 (96.02)	
Black	29 (6.65)	23 (12.43)	6 (2.39)	
Other	9 (2.06)	5 (2.70)	4 (1.59)	
*Charlson–Deyo Score*				0.81
0	270 (61.93)	118 (63.78)	152 (60.56)	
1	82 (18.81)	35 (18.92)	47 (18.73)	
2	31 (7.11)	11 (5.95)	20 (7.97)	
3	53 (12.16)	21 (11.35)	32 (12.75)	
*Facility type*				0.025
Academic	164 (37.61)	71 (38.38)	93 (37.05)	
Non-academic	267 (61.24)	109 (58.92)	158 (62.95)	
Unknown	5 (1.15)	5 (2.70)	0 (0.00)	
*Great circle distance*	11.60 (5.70–29.70)	12.30 (6.35–30.40)	10.95 (5.10–28.20)	0.42
*Urban/Rural*				0.32
Urban	409 (93.81)	173 (93.51)	236 (94.02)	
Rural	11 (2.52)	3 (1.62)	8 (3.19)	
Unknown	16 (3.67)	9 (4.86)	7 (2.79)	
*Insurance*				0.021
Private	95 (21.79)	48 (25.95)	47 (18.73)	
Medicaid	27 (6.19)	9 (4.86)	18 (7.17)	
Medicare	302 (69.27)	119 (64.32)	183 (72.91)	
Unknown	12 (2.75)	9 (4.86)	3 (1.20)	
*T-stage*				0.002
Ta	300 (68.81)	142 (76.76)	158 (62.95)	
Tis	136 (31.19)	43 (23.24)	93 (37.05)	
*Tumor size*				0.34
<2 cm	58 (13.30)	29 (15.68)	29 (11.55)	
≥2 cm	45 (10.32)	21 (11.35)	24 (9.56)	
Unknown	333 (76.38)	135 (72.97)	198 (78.88)	
*Histologic Grade*				0.44
Low	164 (37.61)	76 (41.08)	88 (35.06)	
High	170 (38.99)	69 (37.30)	101 (40.24)	
Unknown	102 (23.39)	40 (21.62)	62 (24.70)	
*Treatment approach*				<0.001
No Subsequent Treatment	44 (10.09)	25 (13.51)	19 (7.57)	
Ablation only	263 (60.32)	106 (57.30)	157 (62.55)	
Urethrectomy	63 (14.45)	39 (21.08)	24 (9.56)	
Ablation + topical	66 (15.14)	15 (8.11)	51 (20.32)	

Data are presented as mean ± SD or median (IQR) for continuous measures, and No. (%) for categorical measures. Chi-square or Fisher’s exact test for categorical variables and *t*-test or Mann–Whitney test for continuous variables were used to compare the two groups.

**Table 2:** Multinomial Logistic Regression Models of Factors Associated with Treatment Selection in Non-Prostatic and Prostatic Noninvasive Urothelial Carcinoma of the Urethra.

	Non-Prostatic Urethra		Prostatic Urethra	
	OR (95% CI)	*p*-Value	OR (95% CI)	*p*-Value
**Urethrectomy**				
*Charlson–Deyo score*				
0	Reference		Reference	
1	1.87 (0.59, 5.97)	0.288	0.49 (0.13, 1.85)	0.294
2	0.87 (0.15, 4.88)	0.87	0.00 (0.00)	0.991
3	1.04 (0.24, 4.46)	0.962	0.46 (0.08, 2.73)	0.391
*Facility Type*				
Academic	Reference		Reference	
Non-academic	0.23 (0.08, 0.60)	0.003	0.21 (0.06, 0.67)	0.008
*T-stage*				
Ta	Reference		Reference	
Tis	3.27 (1.10, 9.69)	0.033	3.36 (1.00, 11.37)	0.051
*Tumor Size*				
<2 cm	Reference		Reference	
≥2 cm	2.33 (0.38, 14.23)	0.36	0.14 (0.02, 1.23)	0.076
Unknown	0.94 (0.22, 3.94)	0.932	0.07 (0.01, 0.36)	0.002
*Tumor Grade*				
Low	Reference		Reference	
High	15.15 (3.82, 60.04)	<0.001	59.29 (4.61, 763.17)	0.002
Unknown	7.43 (1.53, 36.11)	0.013	32.01 (2.32, 441.63)	0.01
**Ablation and Topical**				
*Charlson–Deyo score*				
0	Reference		Reference	
1	0.65 (0.14, 3.02)	0.578	0.33 (0.11, 0.96)	0.042
2	No data		1.55 (0.52, 4.59)	0.427
3	No data		0.22 (0.05, 0.91)	0.036
*Facility Type*				
Academic	Reference		Reference	
Non-academic	0.56 (0.14, 2.28)	0.419	1.40 (0.66, 2.98)	0.382
*T-stage*				
Ta	Reference		Reference	
Tis	2.05 (0.49, 8.53)	0.325	2.53 (1.14, 5.62)	0.023
*Tumor Size*				
<2 cm			Reference	
≥2 cm	1.77 (0.15, 20.49)	0.648	0.35 (0.07, 1.84)	0.213
Unknown	0.78 (0.11, 5.64)	0.804	0.63 (0.20, 2.01)	0.434
*Tumor Grade*				
Low	No data		Reference	
High	NA		3.09 (1.21, 7.90)	0.018
Unknown	NA		2.59 (0.85, 7.83)	0.092

The reference treatment group for each model was endoluminal ablation alone. Odds ratios (ORs), 95% confidence intervals (CIs), and *p*-values are presented for treatment categories: urethrectomy, and ablation combined with topical intraluminal therapy. Separate models were constructed for the non-prostatic and prostatic urethral cohorts. NA indicates the variable was not applicable or not estimable due to sample size constraints. Results for the “no subsequent treatment” category are presented in Supplementary Table S1.

**Table 3:** Temporal Trends in Treatment Modalities Among Patients with Non-Prostatic and Prostatic Urethral Carcinoma (2018–2021).

	Non-Prostatic Urethral Carcinoma					Prostatic Urethral Carcinoma				
	No Subsequent Treatment	Ablation Only	Urethrectomy	Ablation + Topical	*p*-Value	No Subsequent Treatment	Ablation Only	Urethrectomy	Ablation + Topical	*p*-Value
	*n* = 25	*n* = 106	*n* = 39	*n* = 15		*n* = 19	*n* = 157	*n* = 24	*n* = 51	
Year of Diagnosis					0.43					0.35
2018	5 (9.62)	29 (55.77)	14 (26.92)	4 (7.69)		5 (10.42)	34 (70.83)	5 (10.42)	4 (8.33)	
2019	6 (11.76)	30 (58.82)	8 (15.69)	7 (13.73)		5 (6.85)	49 (67.12)	5 (6.85)	14 (19.18)	
2020	6 (14.63)	26 (63.41)	6 (14.63)	3 (7.32)		3 (4.69)	35 (54.69)	7 (10.94)	19 (29.69)	
2021	8 (19.51)	21 (51.22)	11 (26.83)	1 (2.44)		6 (9.09)	39 (59.09)	7 (10.61)	14 (21.21)	
*p*-value	0.96	0.52	0.15	0.27		0.47	0.51	0.69	0.12	

Values are reported as No (%) of patients per year. *p*-values in the horizontal row represent results from Cochran–Armitage tests for trend across years within each treatment group. The overall *p*-value at the top of the table was derived from a Chi-square test comparing the distribution of all treatment modalities across years.

**Table 4:** Multivariable Cox Proportional Hazards Regression for Overall Survival in Patients with Non-Invasive Urethral Carcinoma.

	Non-Prostatic Urethra		Prostatic Urethra	
	HR [95% CI]	*p*-Value	HR [95% CI]	*p*-Value
*Treatment Selection*				
Surveillance	Reference		Reference	
Ablation only	0.85 (0.31, 2.34)	0.755	1.31 (0.47, 3.65)	0.61
Surgical	1.87 (0.78, 4.50)	0.164	1.25 (0.47, 3.30)	0.66
Ablation + topical	1.40 (0.42, 4.62)	0.582	0.18 (0.05, 0.60)	0.005
*Age*	1.03 (0.99, 1.07)	0.203	1.07 (1.03, 1.11)	<0.001
*Race*				
White	Reference			
Black	0.70 (0.19, 2.54)	0.586	1.25 (0.35, 4.47)	0.736
Other	NA		0.90 (0.11, 7.10)	0.917
*Charlson–Deyo Score*				
0	Reference		Reference	
1	2.66 (1.29, 5.45)	0.008	1.92 (0.97, 3.81)	0.062
2	1.79 (0.48, 6.65)	0.384	3.54 (1.49, 8.42)	0.004
3	1.03 (0.33, 3.22)	0.954	2.71 (1.33, 5.49)	0.006
*T-stage*				
Ta	Reference		Reference	
Tis	0.52 (0.23, 1.19)	0.12	1.00 (0.55, 1.82)	0.993
*Tumor size*				
<2 cm	Reference		Reference	
≥2 cm	7.77 (1.26, 47.73)	0.027	1.92 (0.65, 5.66)	0.239
Unknown	6.14 (1.15, 32.68)	0.033	1.32 (0.54, 3.21)	0.543
*Grade*				
Low	Reference		Reference	
High	0.93 (0.42, 2.05)	0.86	1.38 (0.67, 2.85)	0.376
Unknown	0.54 (0.20, 1.43)	0.216	1.40 (0.60, 3.25)	0.433

Multivariable Cox proportional hazards models were constructed separately for non-prostatic and prostatic urethral carcinoma cohorts. Hazard ratios (HRs), 95% confidence intervals (CIs), and *p*-values are reported for each covariate. Reference categories are specified within each variable group. All variables included in prior treatment selection models were retained. “NA” indicates variables not applicable or not estimable due to sample size constraints.

## Data Availability

The data that support the findings of this study were obtained from the National Cancer Database (NCDB), which is jointly sponsored by the American College of Surgeons and the American Cancer Society. Access to the NCDB is restricted and subject to approval by the Commission on Cancer. Additional information is available at: https://www.facs.org/quality-programs/cancer/ncdb (accessed on 13 December 2024).

## Supplementary Materials

The following supporting information can be downloaded at https://www.mdpi.com/article/10.3390/curroncol33010045/s1, Table S1: Complete multinomial logistic regression model for factors associated with treatment selection in non-prostatic and prostatic urethral carcinoma; Table S2: Multivariable Cox Regression for Overall Survival in Non-Invasive Urethral Carcinoma, including all Prespecified Covariates.
