# Supplementary material for: Real-World Treatment Patterns and Outcomes of Intraluminal Ablative Therapies in Noninvasive Urethral Carcinoma: A National Cancer Database Analysis
Source: Curr Oncol. 2026 Jan 14;33(1):45. doi: 10.3390/curroncol33010045 (PMC12840007; doi:10.3390/curroncol33010045)
Supplement: Supplementary file 1 [file curroncol-33-00045-s001.zip › curroncol-4044961-supplementary.pdf]

Supplementary Table S1. Complete multinomial logistic regression model for factors associated with treatment selection in non-prostatic and prostatic urethral carcinoma.

	Non-Prostatic Urethra		Prostatic Urethra	
	OR (95% CI)	P-value	OR (95% CI)	P-value
**No Subsequent Treatment**				
Age	1.01 (0.94, 1.07)	0.875	1.00 (0.95, 1.06)	0.891
Male	0.31 (0.08, 1.20)	0.089	No data	
Race				
White	Reference			
Black	2.79 (0.48, 16.21)	0.252	2.01 (0.16, 25.82)	0.592
Other	1.07 (0.08, 15.13)	0.961	2.39 (0.17, 33.20)	0.516
Charlson-Deyo score				
0	Reference			
1	0.37 (0.08, 1.73)	0.208	No data	
2	0.82 (0.08, 8.67)	0.87	1.12 (0.19, 6.49)	0.896
3	0.92 (0.19, 4.40)	0.912	0.70 (0.15, 3.23)	0.643
Facility Type				
Academic	Reference			
Non-academic	3.08 (0.93, 10.18)	0.065	1.37 (0.42, 4.44)	0.599
Insurance				
Private	Reference			
Medicaid	2.20 (0.14, 34.10)	0.574	1.13 (0.09, 14.72)	0.928
Medicare	3.28 (0.60, 17.88)	0.169	0.77 (0.18, 3.21)	0.714
Unknown	2.56 (0.14, 45.71)	0.522	No data	
T-stage				
Ta	Reference			
Tis	0.37 (0.06, 2.17)	0.272	1.85 (0.54, 6.35)	0.329
Tumor Size				
<2 cm	No data		Reference	
≥2 cm	NA		1.20 (0.06, 23.22)	0.903
Unknown	NA		2.92 (0.33, 25.64)	0.335
Tumor Grade				
Low	Reference		Reference	
High	0.34 (0.09, 1.33)	0.123	0.21 (0.04, 1.08)	0.061
Unknown	0.56 (0.13, 2.36)	0.431	1.13 (0.30, 4.25)	0.86
**Urethrectomy**				
Age	0.97 (0.92, 1.03)	0.358	0.95 (0.89, 1.01)	0.131
Male	2.55 (0.61, 10.60)	0.199	No data	
Race				
White	Reference			
Black	1.79 (0.44, 7.24)	0.411	NA	
Charlson-Deyo score				
0	Reference		Reference	
1	1.87 (0.59, 5.97)	0.288	0.49 (0.13, 1.85)	0.294
2	0.87 (0.15, 4.88)	0.87	0.00 (0.00, .)	0.991
3	1.04 (0.24, 4.46)	0.962	0.46 (0.08, 2.73)	0.391
Facility Type				
Academic	Reference		Reference	
Non-academic	0.23 (0.08, 0.60)	0.003	0.21 (0.06, 0.67)	0.008

Insurance				
Private	Reference		Reference	
Medicaid	1.20 (0.15, 9.46)	0.862	1.14 (0.15, 8.61)	0.899
Medicare	0.73 (0.20, 2.72)	0.639	0.92 (0.21, 4.16)	0.918
T-stage				
Ta	Reference		Reference	
Tis	3.27 (1.10, 9.69)	0.033	3.36 (1.00, 11.37)	0.051
Tumor Size				
<2 cm	Reference		Reference	
≥2 cm	2.33 (0.38, 14.23)	0.36	0.14 (0.02, 1.23)	0.076
Unknown	0.94 (0.22, 3.94)	0.932	0.07 (0.01, 0.36)	0.002
Tumor Grade				
Low	Reference		Reference	
High	15.15 (3.82, 60.04)	<0.001	59.29 (4.61, 763.17)	0.002
Unknown	7.43 (1.53, 36.11)	0.013	32.01 (2.32, 441.63)	0.01
**Ablation and Topical**				
Age	0.98 (0.90, 1.06)	0.642	0.98 (0.94, 1.02)	0.234
Male	1.23 (0.28, 5.49)	0.784	No data	
Race				
White	Reference		Reference	
Black	No data	0.993	No data	
Other	No data		0.97 (0.06, 15.56)	0.985
Charlson-Deyo score				
0	Reference		Reference	
1	0.65 (0.14, 3.02)	0.578	0.33 (0.11, 0.96)	0.042
2	No data		1.55 (0.52, 4.59)	0.427
3	No data		0.22 (0.05, 0.91)	0.036
Facility Type				
Academic	Reference		Reference	
Non-academic	0.56 (0.14, 2.28)	0.419	1.40 (0.66, 2.98)	0.382
Insurance				
Private	Reference		Reference	
Medicaid	2.81 (0.13, 61.22)	0.511	1.89 (0.40, 8.90)	0.422
Medicare	1.37 (0.20, 9.53)	0.747	1.42 (0.49, 4.10)	0.523
Unknown	No data		13.90 (0.57, 341.43)	0.107
T-stage				
Ta	Reference		Reference	
Tis	2.05 (0.49, 8.53)	0.325	2.53 (1.14, 5.62)	0.023
Tumor Size				
<2 cm			Reference	
≥2 cm	1.77 (0.15, 20.49)	0.648	0.35 (0.07, 1.84)	0.213
Unknown	0.78 (0.11, 5.64)	0.804	0.63 (0.20, 2.01)	0.434
Tumor Grade				
Low	No data		Reference	
High	NA		3.09 (1.21, 7.90)	0.018
Unknown	NA		2.59 (0.85, 7.83)	0.092

The reference treatment group for each model was endoluminal ablation alone. Odds ratios (ORs), 95% confidence intervals (CIs), and P-values are presented for three treatment categories: no subsequent treatment, urethrectomy, and ablation combined with topical intraluminal therapy. Separate models were constructed for the non-prostatic and prostatic urethral cohorts. Significant predictors ($P < .05$) are highlighted in bold. NA indicates the variable was not applicable or not estimable due to sample size constraints; “No data” indicates unavailable or suppressed data in the NCDB dataset.

Supplementary Table S2. Multivariable Cox Regression for Overall Survival in Non-Invasive Urethral Carcinoma, including All Prespecified Covariates.

	**Non-Prostatic Urethra**		**Prostatic Urethra**	
	**HR [95% CI]**	**P-value**	**HR [95% CI]**	**P-value**
Treatment Selection				
Surveillance	Reference		Reference	
Ablation only	0.85 (0.31, 2.34)	0.755	1.31 (0.47, 3.65)	0.61
Surgical	1.87 (0.78, 4.50)	0.164	1.25 (0.47, 3.30)	0.66
Ablation+topical	1.40 (0.42, 4.62)	0.582	0.18 (0.05, 0.60)	0.005
Age	1.03 (0.99, 1.07)	0.203	1.07 (1.03, 1.11)	<0.001
Male	0.99 (0.39, 2.47)	0.977	NA (All males)	
Race				
White	Reference			
Black	0.70 (0.19, 2.54)	0.586	1.25 (0.35, 4.47)	0.736
Other	NA		0.90 (0.11, 7.10)	0.917
Charlson-Deyo Score				
0	Reference		Reference	
1	2.66 (1.29, 5.45)	0.008	1.92 (0.97, 3.81)	0.062
2	1.79 (0.48, 6.65)	0.384	3.54 (1.49, 8.42)	0.004
3	1.03 (0.33, 3.22)	0.954	2.71 (1.33, 5.49)	0.006
Facility type				
Academic	Reference		Reference	
Non-academic	1.31 (0.67, 2.55)	0.429	1.01 (0.57, 1.79)	0.984
Unknown	NA			
Insurance				
Private	Reference		Reference	
Medicaid	0.53 (0.06, 4.98)	0.579	0.26 (0.05, 1.51)	0.135
Medicare	1.61 (0.58, 4.46)	0.362	0.68 (0.28, 1.62)	0.379
Unknown	4.97 (0.43, 57.35)	0.199	2.55 (0.27, 24.29)	0.416
T-stage				
Ta	Reference		Reference	
Tis	0.52 (0.23, 1.19)	0.12	1.00 (0.55, 1.82)	0.993
Tumor size				
<2 cm	Reference		Reference	
≥2 cm	7.77 (1.26, 47.73)	0.027	1.92 (0.65, 5.66)	0.239
Unknown	6.14 (1.15, 32.68)	0.033	1.32 (0.54, 3.21)	0.543
Grade				
Low	Reference		Reference	
High	0.93 (0.42, 2.05)	0.86	1.38 (0.67, 2.85)	0.376
Unknown	0.54 (0.20, 1.43)	0.216	1.40 (0.60, 3.25)	0.433

Multivariable Cox regression models were constructed separately for the non-prostatic and prostatic urethral carcinoma cohorts. Hazard ratios (HRs), 95% confidence intervals (CIs), and *P*-values are presented for each covariate. Reference categories are indicated within each variable grouping. "NA" denotes variables that were not applicable or not estimable due to sample size limitations. "Unknown" indicates missing or unreported data within the NCDB dataset.
